# Individual differences in sensory and expectation driven interoceptive processes: a novel paradigm with implications for alexithymia, disordered eating and obesity

**DOI:** 10.1038/s41598-021-89417-8

**Published:** 2021-05-12

**Authors:** Hayley A. Young, Chantelle M. Gaylor, Danielle de-Kerckhove, David Benton

**Affiliations:** Department of Psychology, Swansea University, Wales, SA2 8PP UK

**Keywords:** Physiology, Psychology

## Abstract

Those with disordered eating and/or obesity often express difficulties in sensing or interpreting what is happening in the body (interoception). However, research is hindered by conceptual confusion, concerns surrounding domain specificity, and an inability to distinguish sensory (bottom-up) and expectation driven (top-down) interoceptive processes. A paradigm was therefore developed from an active inference perspective. Novel indices were computed and examined in those with alexithymia: a personality associated with interoceptive deficits and disordered eating. The paradigm successfully identified individuals driven by sensations rather than expectations: alexithymia was characterized by attenuated prior precision (a larger divergence between pre-prandial and post-prandial satiety, and low expectation confidence), and increased prediction error (a higher correlation between changes in hunger and blood glucose, and greater rebound hunger after a sensory incongruent drink). In addition, those with a higher BMI were less confident and had a larger anticipated satiety divergence. These findings demonstrate the need to move beyond existing paradigms such as the Satiety Quotient and Heartbeat Counting Task which may have limited our understanding of eating behaviour.

## Introduction

Disordered eating and obesity significantly increase the risk of morbidity and mortality^1^, yet despite decades of scientific research, interventions have met with limited success. Therefore, a deeper understanding of the mechanisms driving these behaviours is required. Interoception (the process of detecting and interpreting bodily sensations) may be critical to understanding eating behaviour ^2^. However, recent advances in our understanding of brain functioning ^3^ has revealed that existing interoception paradigms tend to confound sensory and belief driven processes. Therefore, taking an active inference perspective (an extension of the predictive coding framework) we developed an ecologically valid interoception paradigm specifically concerning satiety (the feeling of fullness and the suppression of hunger for a period after consumption). The paradigms efficacy was established by examining as an exemplar a sub-component of alexithymia, the difficulty in identifying feelings (DIF). Alexithymia is a sub-clinical trait characterised by difficulty identifying feelings and somatic sensations (DIF), difficulty describing feelings (DDF), and an externally orientated thinking style (EOT) ^4^. Here we focused on DIF as this component is highly prevalent amongst those with disordered eating ^5^. Importantly, taking this approach we found that those with DIF lacked confidence in their satiety expectations rather than sensory sensitivity. Consequently, in those with DIF, postprandial satiety relied predominantly on changes in physiology, rather than on prior expectations. To the best of our knowledge, this is the first time individual differences in processes contributing to satiety have been elucidated.

Interoception has been of interest to researchers in various fields, including obesity ^6,7^, eating disorders ^8^, and emotion ^9^, however, individual differences have been notoriously difficult to quantify. As a result, the supposed associations between interoception and eating behaviour rely mostly on self-evaluation ^10–12^. Although this dimension of interoception is potentially important, it relies on participants having accurate insight into their abilities, something they often lack ^2,13,14^. Importantly, a variety of objective methods for measuring interoceptive abilities have been proposed including: (1) The Heartbeat Counting Task (HCT) ^15^, which involves participants counting their heartbeats over a specified epoch: their count is then compared to their actual number of heartbeats to produce an interoceptive accuracy score, (2) The Satiety Quotient (SQ) ^16^, where participants judge the degree of a subjective state (e.g., hunger or fullness) before and after consuming a test meal ab libitum—the SQ is calculated by taking the post-eating rating away from the pre-eating rating and dividing by the amount of ad libitum calories consumed ^16^, and (3) The Water Load Test (WLT) ^17^, which involves participants drinking non-carbonated water until reaching the point of perceived satiation, and then again until reaching the point of maximum fullness. In the WLT the percentage of total volume required to reach satiation is used as an index of gastric interoception. Although these tasks have contributed to our understanding of interoception and disordered eating, they have given rise to inconsistent results ^2,8,18–22^, which might reflect their methodological limitations ^23^.

For example, using the HCT some studies have reported that performance is impaired in those with eating disorders ^8,18–20^. However, other studies have found opposite or null effects ^2,19,21,22^. Similar inconsistent findings exist in the relationship between heartbeat counting and DIF ^23–28^. In the context of eating behaviour, an obvious limitation is the questionable relevance of the HCT. As it was reported that heartbeat perception was associated with emotional eating but not to other eating styles^2^, HCT performance may only be relevant to the emotional aspects of disordered eating. Additionally, the effect size of correlations between measures of gastric and cardiac interoception are moderate ^29^, suggesting that poor HCT performance may not equate to interoceptive deficits in other modalities.

**Figure 1:**
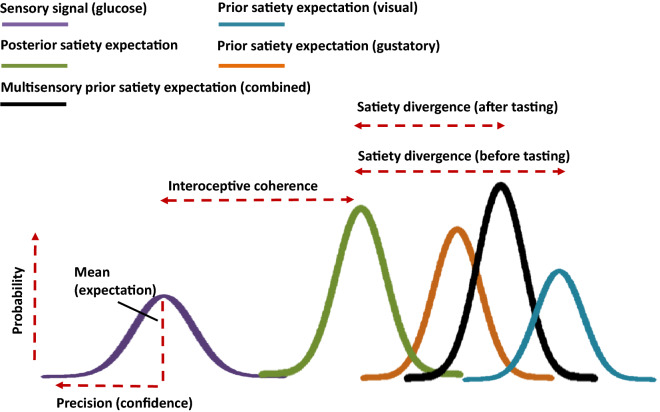
A schematic illustration of the present paradigm in the context of precision weighted inference. Under the principles of Bayesian inference and predictive coding, “top-down” predictions and “bottom-up” prediction errors are weighted by their precision or statistical confidence (i.e. inverse variance). Here visual and gustatory signals are combined to form an initial posterior satiety expectation: a high precision weighting of the gustatory signal (orange), relative to that of the visual signals (blue), enhances the gustatory influence on the posterior expectation (black). Note that as perception is a temporal process, Bayesian inference is reiterated as information from each measurement is added to the knowledge gained from all previous measurements—therefore this initial posterior expectation (black) will subsequently become the prior satiety expectation in anticipation of the next sensory input (i.e., how satiated I will feel once I have consumed this drink). In this example, this new multisensory prior (black) is precise, relative to the new interoceptive information (purple i.e., changes in blood glucose), and predominates subsequent perception (the updated posterior expectation: green i.e., how satiated I now feel). Therefore, the interoceptive coherence index (correlation between postprandial changes in blood glucose and hunger) indicated the degree to which individuals relied on interoceptive information in guiding their perception. Conversely, the satiety divergence index (absolute difference between expected and actual satiety) indicted the degree to which participants integrated prior beliefs.

Perhaps more importantly, recent models of brain functioning indicate that the aforementioned interoception paradigms may need refining due to their tendency to confound expectations and sensory driven processes. It was argued that interoceptive processes fractionate into those driven by top-down expectations, and those caused by bottom-up sensations ^3,30–33^. Accordingly, perceptual (including interoceptive) representations are constructed by assimilating prior expectations, and new information concerning the present interoceptive state ^33^. An implication is that there will be individual variation in the way appetitive representations are constructed. Some individuals may rely primarily on prior beliefs, while others will depend on incoming sensory evidence. The relative precision (confidence) afforded to expectations and sensations will determine their degree of influence (Fig. 1). Unfortunately, current interoception paradigms are unable to distinguish the relative contribution of bottom-up and top-down interoceptive processes. For example, there is evidence that manipulating beliefs about heart rate influences participants’ reports of the number of beats they felt during the HCT ^34,35^. The SQ ^16,36^, is influenced by both changes to the internal milieu ^6^, and expectations about satiety ^37^, and although the volume of consumed water is disguised in the WLT^17^, expectations associated with the gustatory properties of water remain.

An extension of this concern is that current interoception paradigms rely predominantly on retrospective judgements. Participants are often asked to reflect on how they felt previously, or to react according to how they feel right now. For example, “how many heartbeats did you count” or “how hungry are you feeling right now”. In the WLT individuals are asked to “concentrate on their current abdominal sensations, especially if their stomach felt full or empty” ^17^. However, by its nature interoception is a prospective regulatory process. This process is detailed in recent formulations of active inference ^32^, and is critical to explaining how motivated actions facilitate prospective energy regulation ^38^. Essentially, the brain must not only infer the cause of present sensations, but also anticipate how, depending on potential actions, interoceptive signals might change in the future. Therefore, individuals may also vary in their certainty about the future interoceptive consequences of consuming particular foods. For example, in relation to eating behaviour individuals may differ in their confidence about how full they will feel after they have consumed the same food/drink (e.g., satiety expectation confidence ^39^). Therefore, it is important that interoception paradigms are able to capture the anticipatory nature of interoceptive beliefs—there is a need to move towards *prospectively* assessing interoception. Although attempts have been made (e.g., expected satiety ^40^), to date such approaches have not been applied to understanding individual differences.

**Figure 2:**
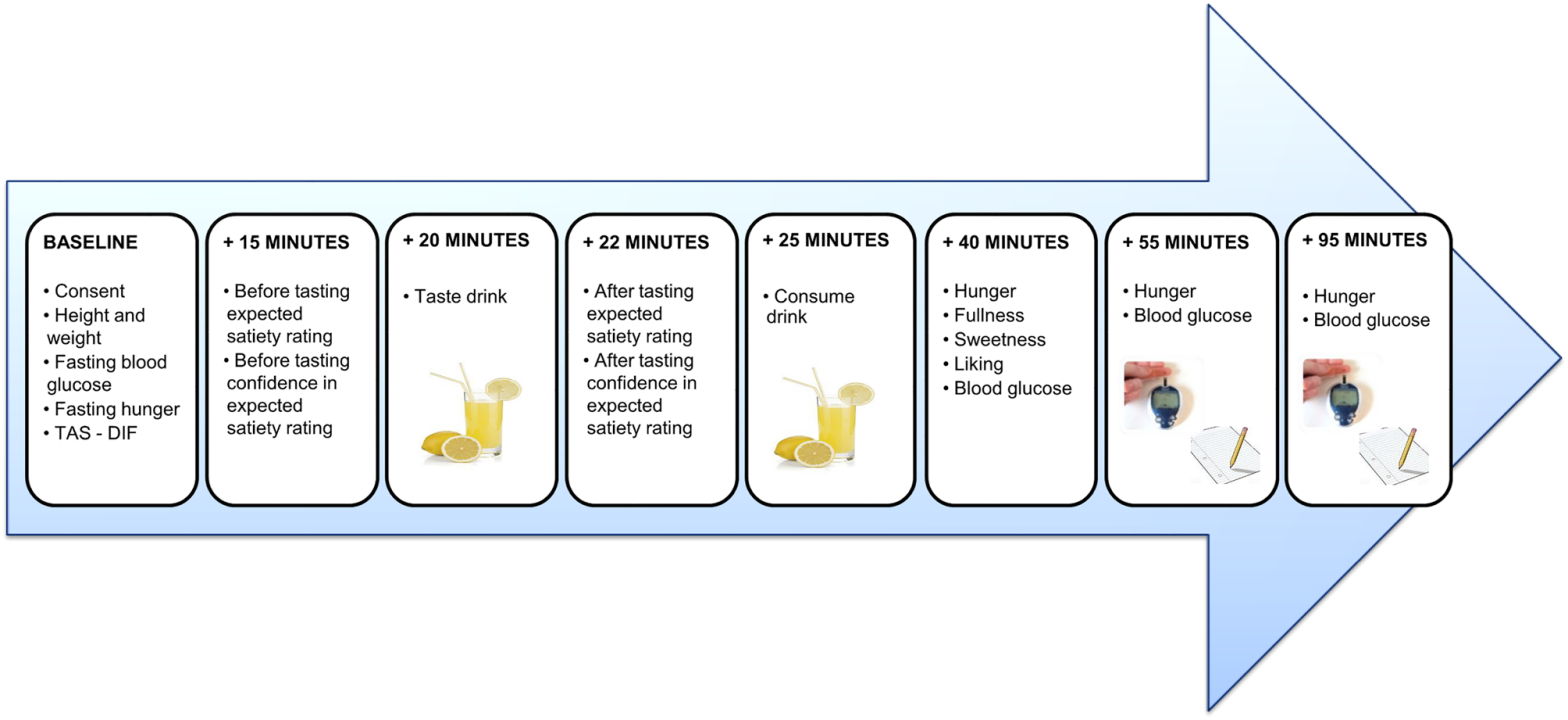
The experimental procedure. *TAS* Toronto Alexithymia Scale, *DIF* difficulty identifying feelings.

**Figure 3:**
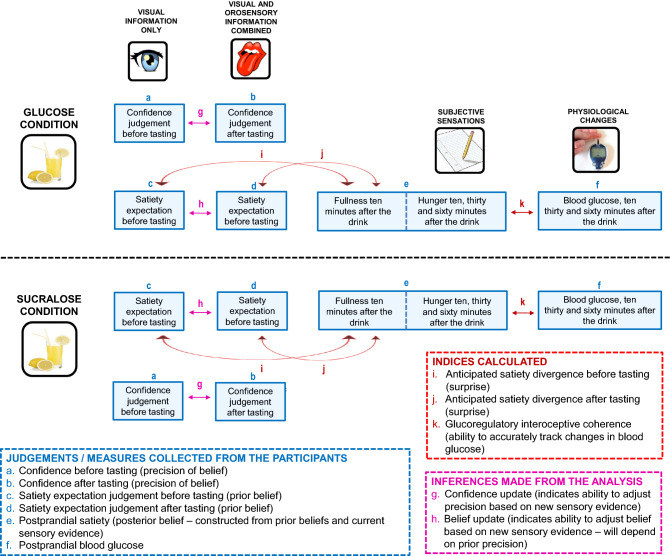
A schematic illustration of the procedure and the derived interoceptive indices.

In this context, the aim of the present study was to develop an interoception paradigm that met the following criteria. First, the paradigm should assess interoception in an eating relevant domain. Second, it should be able to identify individual differences in the propensity to rely on expectations rather than sensations. Third, the paradigm should assess interoceptive beliefs prospectively rather than retrospectively. To achieve these goals, we measured individuals’ satiety expectations and satiety responsivity to drinks varying in flavour/nutrient congruity, while simultaneously monitoring changes in physiology. This allowed the calculation of a number of indices that reflected the relative precision (confidence) of both prior beliefs and sensory evidence (Figs. 1, 2, 3). The efficacy of the paradigm was assessed by examining the pattern of relationships between these indices from an active inference perspective, and by relating these indices to differences in DIF. Crucially, evidence is provided that the approach allowed us to determine the relative contribution of prospective expectations and incoming sensory signals in an interoceptive domain relevant to eating behaviour.

## Methods

### Procedure

Swansea University Department of Psychology ethics committee approved this procedure, which was conducted in accordance with the principles laid down by the declaration of Helsinki 2013. Participants fasted for a minimum of 8 h before attending the laboratory. After providing their written informed consent, participants had their height, weight and fasting blood glucose measured, rated their fasting hunger, and completed the questionnaire pack detailed below. The participants were then allocated to receive either glucose (flavour/nutrient congruent) or sucralose (flavour/nutrient incongruent). Note that the difference between the flavour-nutrient congruent and flavour-nutrient incongruent drinks was the nutrient content rather than the flavour. The random sequence was computer generated by HY who produced the solutions in sequentially numbered tumblers. Participants were then allocated double blind by CG. To reduce variability, participants were matched on DIF (Glucose M = 16.58, SD = 5.45; Sucralose M = 16.87, SD = 5.82). A repeated measures design was not used because pilot data showed very strong order effects which varied with the level of DIF—a potential consequence of the hypothesised differences in learning rate (i.e., there was the very strong potential that DIF would interact with the order of presentation of the drinks).

After being assigned their drink, participants were asked just to look at the drink and rate the drinks expected satiety and their confidence in these ratings. They then took one sip of the drink and completed the ratings a second time. Participants then had five minutes to consume the entire beverage, before they evaluated how sweet they found the drink, how much they liked the drink, its visual appeal, its mouth-feel, odour, initial taste, and after taste. Ten minutes after consuming the drink (15 min after they started drinking), participants evaluated how full they felt at that moment, and provided another glucose measurement. After 30 and 60 min, blood glucose and hunger were assessed for a third and fourth time.

### Test drinks

Each drink was 500 ml provided in a clear plastic tumbler. The glucose drink contained 75 g of glucose dissolved in water. The sugar free beverage was sweetened with sucralose to produce a similar sweetness to the glucose drink. The two drinks contained 10 ml of lemon juice to increase palatability. An important consideration was that when studying satiety, a number of food properties need to be taken into consideration, such as taste, palatability, energy density, food texture, and perceived volume. These components can be broadly categorised as influencing either *metabolic satiety* (neural and hormonal signals that are transported from the gastrointestinal tract to the brain), or *sensory satiety* (a conditioned response based on previous experience with food whereby the sensory aspects of a food such as appearance, taste etc. immediately conveys information about its satiety value) ^41^. With this in mind the two drinks were designed to be identical in terms of their visual, orosensory, and volumetric properties, differing only in their energy content. This enabled us to determine those individuals most sensitive to postprandial changes in metabolism, in this case blood glucose levels, and those individuals who instead rely on expectations associated with the sensory aspects of a food, in this case the drinks visual and orosensory properties. Given the complexities of the postprandial milieu, we chose to focus on a simple dietary manipulation involving only one macronutrient ^42^. Additionally, as food familiarity provides the basis for satiety expectations ^43^, we selected a food which would be highly familiar to participants i.e., a sweet tasting beverage. Crucially, the sensory match of these drinks had been confirmed during previous research ^44,45^, and was confirmed in the present sample (Supplementary Information—manipulation check).

### Expected satiety

Participants were asked to answer the following questions after seeing and tasting the drink, using 100 mm visual analogue scales (VAS) anchored by ‘Extremely’ and ‘Not at all’. ‘To what extent do you think the drink would fill you up?’ and ‘Are you confident about the extent to which the drink would fill you up?’ VAS were previously shown to be sensitive to detecting differences in expectations between foods ^46^.

### Fullness and hunger

Participants were asked to describe the way they felt ‘at that moment’ by answering the following questions, using 100 mm visual analogue scales (VAS) anchored by ‘Extremely’ and ‘Not at all’. ‘How full do you feel right now?’ and ‘How hungry do you feel right now’?

### Perceived sweetness and liking

Participants were asked to evaluate the sweetness and their liking for the drink: ‘How sweet did you find the drink?’ (Extremely sweet/Not at all sweet) and ‘How much did you like the drink?' (Very much/Not at all).

### Blood glucose

Blood glucose was monitored from finger pricks using an ExacTech sensor (Medisense Britain Limited) that using an enzymic method, coupled with microelectronic measurement, which has been shown to be accurate ^47^.

### Participants

This study included sixty-two females between 18 and 30 years of age that were recruited from the local area through posters on university notice boards and online adverts on popular social media websites (e.g., Facebook). The sample size was based on previous research that has examined: (1) satiety expectation confidence ^39^, (2) glucose and interoception ^48^, (3) the Satiety Quotient ^49^, (4) interoception and eating behaviour ^2^, (5) DIF and interoception ^9^, and (6) individual subjective responsivity to glucose ^44^. Exclusion criteria included any metabolic or cardiovascular disorder, gastrointestinal problems, pregnancy, a current diagnosis of a mood or eating disorder, and/or if they were taking medications or herbal supplements to manage body weight or control appetite. BMI ranged from 18.5 to 38.3  (average 24.34) kg/m^2^—64.5% had a normal BMI between 18.5 and 24.9, 27.4% were overweight with a BMI between 25 and 30, and the remaining 8.1% of the sample were obese with a BMI > 30. BMI was evenly distributed across the glucose and sucralose conditions: X^2^ = 0.259, *p* > 0.879. Participants were instructed to refrain from drinking alcohol and taking part in any physical activity within 24 h of the study and to abstain from consuming any food and drink for at least 8 h before attending the laboratory. All participants completed the study in the morning between 9.00 am and 1.00 pm.

### Toronto alexithymia scale  (TAS-20)

The 7-item Difficulty Identifying Feelings (DIF) ^4^ subscale was used to assess the ability to identify feelings and differentiate between feelings and bodily sensations (the component of alexithymia most often associated with disordered eating ^5,50,51^). Participants indicated their responses to statements (e.g., “I am often confused about what emotion I am feeling”) by selecting the answer that best describes them on a five-point scale (1 = strongly disagree to 5 = strongly agree). Higher scores indicate more difficulty identifying feelings. Internal consistency in the current study was high (α = 0.86).

### Body mass index (BMI)

An objective measure of body weight was taken using an accurate electronic scale (Kern KMS-TM, Kern and Sohn GmbH, Germany) that took 50 assessments over a 5 s period and produced an average value. A stadiometer was used to measure height. The formula weight (kg) /height (m^2^) was used to calculate BMI.

## Interoceptive indices, data preparation and analysis

A number of indices were calculated and used to indicate the relative precision (confidence) of prior beliefs and sensory evidence (prediction error) (Fig. 3).

### Post-prandial hunger and satiety (Fig. 3e)

Postprandial hunger and satiety were considered posterior beliefs (i.e., those made by assimilating expectations and incoming sensations).

### Anticipated satiety divergence (SD) (Fig. 3i,j)

An index of *anticipated satiety divergence* (SD) was calculated by taking the difference between expected satiety and actual satiety 10 min after consuming the drink. Two scores were calculated using expected satiety before (visual information only) (Fig. 3c) and after tasting (visual and gustatory information) (Fig. 3d). Hereafter these scores are referred to as satiety divergence before tasting (SD-BT) (Fig. 3i) and satiety divergence after tasting (SD-AT) (Fig. 3j) the drink. These indices were calculated using the following transformation: 1- ( (absolute (expected satiety − actual satiety))/ (expected satiety + actual satiety)). Therefore, a higher score indicated a lower divergence between the participants pre-prandial and post-prandial satiety—these individuals would be driven primarily by their expectations. Conversely, individuals with a lower score had a larger divergence between their pre-prandial and post-prandial satiety implying that changes in their interoceptive signals were unexpected—these individuals tend not to integrate expectations into their beliefs about the current interoceptive state.

### Interoceptive coherence (IC) (Fig. 3k)

From an active inference perspective, the regulation of internal states is largely subconscious. However, afferent sensations affording high precision may be more likely to be associated with subjective symptoms (e.g., hunger) ^45,52,53^. Therefore, we used the within person correlation coefficient between blood glucose values (0, 15, 30, and 60 min) (Fig. 3e) and hunger (0, 15, 30, and 60 min) (Fig. 3f) as an index of sensory sensitivity within the glucose domain i.e., the degree to which individuals integrate afferent information into their beliefs about the current interoceptive state (*interoceptive coherence* (IC)) (Fig 3k). Note that this variable was reversed so that a higher score indicated that blood glucose tracked hunger more coherently—these individuals would be primarily driven by unfolding sensations. Unexpectedly some participants, mostly in the sucralose condition, reported increasing hunger despite increasing blood glucose levels (i.e., a positive correlation, rather than the expected negative correlation, between blood glucose and hunger). As both a high negative coefficient and a high positive coefficient indicate a strong linear relationship between blood glucose and hunger (i.e., coherence), the absolute correlation coefficient was also considered as a Supplementary Analysis (Supplementary Table S1). Although effect sizes differed, overall this supplementary analysis gave rise to a similar pattern of results to those reported below using the signed correlation.

From an active inference perspective, we would expect SD and IC scores to be inversely related. In other words, those driven primarily by expectations (high SD) should be less sensitive to changes in postprandial blood glucose levels (low IC). Conversely, those driven by sensations (high IC) should be less likely to integrate prior expectations into their subsequent satiety (low SD) (Fig. 1). Thus, together SD and IC give an indication of the degree to which individuals rely on prospective beliefs as opposed to incoming sensory evidence when making inferences about their current interoceptive state.

### Expected satiety confidence (ESC) (Fig. 3a,b)

It has been argued previously that the precision of conscious beliefs might be experienced as subjective confidence ^54^. Therefore, *expected satiety confidence* (ESC), i.e., participants confidence ratings before and after tasting, provided a further indication of the precision of participant’s beliefs after visual inspection, and combined visual and gustatory information ^54^ (Fig. 3a,b). In addition, the difference between the two confidence measurements indicated the degree to which the participant updated the precision of their belief based on receiving new gustatory evidence (Fig. 3g). Similarly, the difference between expected satiety ratings before and after the drink indicated the degree to which the participant updated their belief based on receiving new gustatory information (Fig. 3h). From an active inference perspective, individuals with more precise prior beliefs before tasting would be less likely to update those beliefs based on new information.

### Rebound hunger

An indication of prediction error responsivity was achieved by capitalising on observations that when a “sensory incongruent” food such as sucralose is consumed (i.e., a sweet taste providing no energy), the flavour-nutrient ‘mismatch’ frequently results in rebound hunger ^55^. Therefore, experimentally manipulating flavour/nutrient congruity allowed us to examine individual differences in the sensitivity to unpredictable interoceptive states. Specifically, by examining individual differences in post-prandial hunger ratings 15, 30 and 60 min after the sucralose drink, compared to the glucose drink, we were able to determine the degree to which an individual’s appetite was driven by their internal state, rather than by expectations associated with the sweet taste (Fig. 3e). We would expect that those driven by incoming sensations would be more responsive to having consumed a drink containing no energy, and therefore having experienced the greatest levels of postprandial hunger. Conversely, individuals who rely on their prior expectations associated with the sweet taste would discount their surprising interoceptive state and maintain an “illusionary” feeling of satiety.

### Glucose tolerance

Finally, from an active inference perspective ‘action’ (including autonomic action involved in the regulation of blood glucose) requires the temporary attenuation of incoming signals which can only occur in the context of precise prior beliefs ^56^. Consequently, if at an subconscious level, beliefs about the optimal level of blood glucose are imprecise, homeostatic regulation will be compromised. Therefore, differences in the change in blood glucose levels after the glucose drink were taken to indicate the precision of subconscious prior beliefs (Fig. 3f).

### Validity of the paradigm

**Figure 4:**
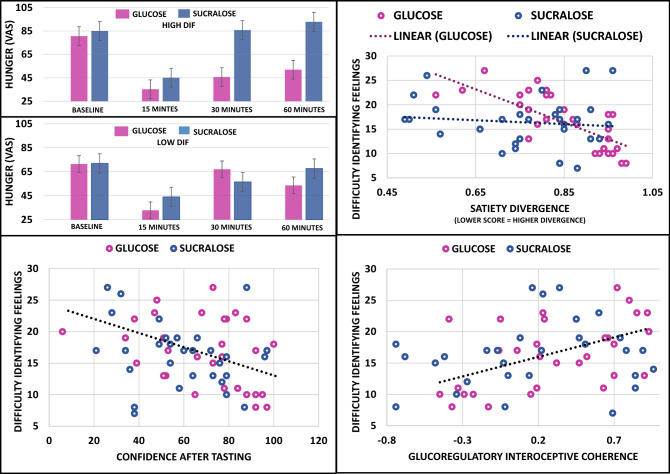
The effect of difficulty identifying feelings on satiety divergence, hunger, confidence and interoceptive coherence. N = 62. Top left: After 30 (p < 0.05) and 60 (p < 0.01) minutes those who consumed sucralose were hungrier but only if they were high in difficulty identifying feelings. Top right: In the glucose condition those high in difficulty identifying feelings were significantly less accurate at anticipating their satiety (greater divergence) (*r* =  − 0.770, p < 0.0001), however, there was no effect in the sucralose condition (*r* = 0.091, p = 0.626). Bottom left: After tasting those high in difficulty identifying feelings were less confident (*r* =  − 0.342, p < 0.006) in their satiety expectation irrespective of whether they tasted glucose or sucralose. Bottom right: Across both conditions those high in DIF had a higher glucoregulatory interoceptive coherence (*r* = 0.258, p < 0.043).

Two lines of evidence concerning the validity of the paradigm were examined. First, the pattern of associations between the interoceptive indices were examined from an active inference perspective (Tables 1, 2). Second, whether the paradigm could decipher expectation versus sensory driven interoceptive processes was established by examining associations with DIF (Fig. 4). Finally, we considered whether the interoceptive indices varied according to BMI (Table 1).

**Table 1:** Zero order correlations between the interoceptive indices, alexithymia, and BMI in the glucose and sucralose conditions.

Glucose							Sucralose						
	ESC-AT	SD-BT	SD-AT	IC	DIF	BMI		ESC-AT	SD-BT	SD-AT	IC	DIF	BMI
ESC-BT	0.503*	0.407*	− 0.027	− 0.263	− 0.123	− 0.108	ESC-BT	0.428*	0.267	− 0.096	− 0.260	0.276	0.053
ESC-AT		0.482**	0.550**	− 0.484**	− 0.370*	− 0.503**	ESC-AT		0.114	0.280	− 0.268	− 0.325*	− 0.441*
SD-BT			0.604**	− 0.313*	− 0.671**	− 0.328	SD-BT			0.386*	− 0.245	0.249	− 0.094
SD-AT				− 0.320*	− 0.713**	− 0.463**	SD-AT				− 0.164	− 0.094	− 0.229
IC					0.499*	0.044	IC					− 0.073	0.410*
DIF						0.360*	DIF						0.367*

N = 62.

*IC* interoceptive coherence (this measure was reversed so that a higher score indicated that as blood glucose levels increased hunger decreased), *SD* satiety divergence (a higher score indicated a lower divergence between the participants pre-prandial and post-prandial satiety − a higher score means individuals would be driven primarily by their expectations), *ESC* expected satiety confidence, *BT* before tasting, *AT* after tasting, *DIF* difficulty identifying feelings, *BMI* body mass index.

Bold—significant positive correlation, Italic—significant negative correlation.

*p < 0.05, **p < 0.001.

**Table 2:** Mean difference (se) between the interoceptive indices in the glucose and sucralose conditions.

	Glucose	Sucralose	95% Confidence interval
ESC-BT	60.90 (4.84)	53.7 4 (4.63)	− 6.25, 20.57
ESC-AT	69.29 (4.01)	58.45 (3.90)	− 0.36, 22.04
SD-BT	1.01 (0.04)	0.97 (0.05)	− 0.09, 0.18
SD-AT	**1.08 (0.04)**	**0.89 (0.05)**	**0.06, 0.32**
IC	− 0.70 (0.07)	− 0.83 (0.09)	− 0.10, 0.37
AUCi for hunger	** − 1606.29 (239.33)**	** − 585.88 (227.57)**	** − 1681.02, − 359.78**

N = 62.

*IC* interoceptive coherence, *SD* satiety divergence, *ESC* expected satiety confidence, *BT* before tasting, *AT* after tasting, *AUCi* area under the curve with respect to ground.

Bold—significantly different.

### Statistical analyses used to test the paradigm

All data were analysed using SPSS (Version 25.0, SPSS, Chicago, IL). Zero order correlations (Pearson’s) were used to examine the pattern of association between interoception indices. Independent t tests determined the group-based effects of manipulating flavour/nutrient congruency. To examine the effect of confidence on satiety expectation updating (Fig. 3h), a repeated measures ANCOVA was conducted: Taste (Before tasting/After tasting) was the repeated measures factor, Drink (Glucose/Sucralose) was the between subjects’ factor, and expected satiety confidence before tasting was the covariate. Raw satiety expectation scores (before and after tasting) were dependant variables.

To determine whether those with DIF differed in terms of their satiety divergence (SD) (Fig. 3i, j) a 2 × 2 repeated measures ANCOVA was conducted: Taste (SD before tasting/SD after tasting) was the repeated measures factor, Drink (Glucose/Sucralose) was the between subjects’ factor, and DIF was the covariate. Where significant interactions resulted, bivariate correlation analysis (Pearson’s r) assessed associations between SD and DIF. Similarly, to examine the effects on confidence, a 2 (ESC before tasting/ESC after tasting) × 2 (Glucose/Sucralose) × DIF repeated measures ANCOVA was conducted. These analyses determined (1) whether those high in DIF differed in the precision of their prior expectations (Fig. 3a,b), and (2) whether they differed in their ability to adjust prior precision given new contextual sensory information (Fig. 3g). Again, bivariate correlation analysis assessed associations between confidence and DIF where interactions were significant. A univariate ANOVA determined whether interoceptive coherence (IC) (Fig. 3k) was associated with DIF depending on the nature of the drink: Drink (Glucose/Sucralose) was the between subjects’ factor, and DIF was the covariate. Bivariate correlation analysis probed significant interactions. A repeated measures ANCOVA determined individual differences in IPE responsivity: Hunger (baseline, 15 min, 30 min, 60 min) (Fig. 3e) was the repeated measures factor, Drink was the between subjects’ factor (Glucose/Sucralose), and DIF was the covariate. Finally, to examine whether those with DIF had poorer glucose tolerance, this analysis was repeated for blood glucose levels (Fig. 3f).

### Assumptions, control of outliers and the proportion of type 1 errors

When tests involved between group comparisons Levene’s test was used to assess the equality of variances and Box’s M was used to determine whether the covariance matrices are similar. All variables met the assumption of normality except SD which was transformed using an arcsine transformation prior to analysis. Cooks distance, with a threshold of N/4, was used to detect possible outliers ^57^. This resulted in two cases, with a Cook’s distance > 0.2, being removed from the SD variable and replaced with the mean. The potential of detecting false positives was controlled using Benjamini and Hochberg’s false discovery rate (FDR) ^58^. The FDR was controlled at *δ* = 0.05. Where significant interactions did not reach this threshold, it is indicated in the text.

## Results: the pattern of association between indices

Zero order correlations between the interoceptive indices are presented in Table 1. In the glucose condition, there was a trend for IC to be inversely related to ESC before tasting (*r* =  − 0.263, *p* = 0.076). In addition, significant negative associations were observed between IC and ESC after tasting (*r* =  − 0.484, *p* < 0.003), SD before tasting (*r* =  − 0.313, *p* < 0.043), and SD after tasting (*r* =  − 0.320, *p* < 0.040). These findings suggested that in relation to a sensory congruent drink, an individual who scores highly on IC is likely to score low on SD and ESC. Although the pattern was similar in the sucralose condition, effect sizes were smaller such that they just missed significance: ESC before tasting (*r* =  − 0.260, *p* = 0.079) and SD before tasting (*r* =  − 0.245, *p* = 0.092), ESC after tasting (*r* =  − 0.268, *p* = 0.072) and SD before tasting (*r* =  − 0.164, *p* = 0.189).

To examine whether more confident individuals were less likely to update their satiety expectation a 2 (Before tasting/After tasting) × 2 (Glucose/Sucralose) ANCOVA with before tasting ESC as the covariate was conducted. The dependant variables were satiety expectations before and after tasting (raw scores). There was a main effect of taste (*F* (1, 58) = 11.395, *p* < 0.001, η^2^ = 0.164). Participants thought the drink would be more filling after tasting (before 51.90 (2.85), after 55.06 (3.14)) but as the drinks were designed to be equally sweet this did not depend on the nature of the drink: Taste × Drink interaction (*F* (1, 58) = 0.045, *p* = 0.833, η^2^ = 0.001). There was also a Taste × Confidence interaction (*F* (1, 58) = 9.719, *p* < 0.003, η^2^ = 0.144) which indicated that those who were more confident in their beliefs before tasting, had a smaller change in their expected satiety as a result of tasting the drink (*r* =  − 0.336, p < 0.008). The Taste × Drink × Confidence interaction was not significant (*F* (1,58) = 0.219, *p* = 0.641, η^2^ = 0.004); an unsurprising finding given that the drinks were designed to have an identical taste and differ only in terms of their post-ingestive consequences.

To determine the group-level effects of manipulating flavour/nutrient congruence an independent *t* tests determined whether ESC, SD, IC, and postprandial hunger (area under the curve with respect to ground (AUCi)) differenced depending on the drink consumed (Table 2). There were no differences between the glucose and sucralose condition for ESC before or after tasting, SD before tasting or IC (Table 2). However, after tasting participants had a larger discrepancy between their expected and actual satiety (*t* = 2.993, *p* < 0.004) when sucralose was consumed. Additionally, when sucralose was consumed participants reported being hungrier during the postprandial period (*t* =  − 3.09, *p* < 0.003). Importantly, at the end of the study when asked what drink they consumed, the participants were unable to consciously identify the nature of the drink (χ^2^ = 0.259, *p* = 0.879).

### Summary

After tasting glucose, interoceptive coherence was inversely related to expected satiety confidence, and the divergence between pre-prandial and post-prandial satiety (Table 1). In addition, participants who were more confident in their satiety expectation were less likely to update this expectation given new gustatory information. These findings suggested that the present approach might be useful for capturing information concerning satiety from an active inference perspective (see also Fig. 1 for an illustration of how these indices might relate to Bayesian inference and predictive coding). However, on occasion many effect sizes were smaller than anticipated, and thus just missed significance (Table 1). This was particularly the case after artificial sweetener. The appetite system is complex and involves the integration of various neural, metabolic and hormonal signals, many of which remain to be fully elucidated. Therefore, it is plausible that in the context of the large prediction error associated with the consumption of artificial sweetener, individuals may preferentially weight other sources of information e.g., gastric distention, when judging their satiety (see main discussion).

## Results: interoceptive processes in those with alexithymia (DIF)

### Satiety divergence (SD)

A Taste (before/after) × Drink (glucose/sucralose) × DIF (covariate) repeated measures ANCOVA tested whether those with DIF differed in their satiety divergence before and after tasting the drink. The Taste × Drink × DIF interaction did not reach significance (*F* (1, 58) = 2.641, *p* = 0.110, η^2^ = 0.044), and neither did the Taste × DIF interaction (*F* (1, 58) = 1.114, *p* = 0.296, η^2^ = 0.019). Interestingly, the Drink × DIF interaction did reach significance (*F* (1, 58) = 15.302, *p* < 0.0001, η^2^ = 0.209): in the glucose condition, those high on DIF were had a higher divergence (*r* =  − 0.770, *p* < 0.0001); an effect absent in the sucralose condition (*r* = 0.091, *p* = 0.626) (Fig. 4: top right). This result suggests that those high in DIF rely to a lesser extent on prior expectations when asked to report their postprandial appetitive state. Furthermore, this finding supports the use of the satiety divergence index to determine individual differences in prior precision when related to satiety.

### Expected satiety confidence (ESC)

The above analysis was repeated using confidence before and after tasting as the dependant variable. The Taste X Drink X DIF interaction was not significant (*F* (1, 58) = 2.034, *p* = 0.159, η^2^ = 0.034). However, the Taste X DIF interaction reached significance (*F* (1, 58) = 9.750, *p* < 0.003, η^2^ = 0.144). Irrespective of the nature of the drink, DIF was not associated with confidence before tasting (*r* = 0.074, *p* = 0.566). However, after tasting those high in DIF were less confident (*r* =  − 0.342, *p* < 0.006) (Fig. 4: bottom left). In addition, those high in DIF were less likely to update their confidence levels when provided with new gustatory information (confidence after tasting minus confidence before tasting) (*r* =  − 0.385, *p* < 0.002) (the negative correlation indicates that those low in DIF became more confident after tasting the drink, whereas those high in DIF had no change). This effect is further illustrated in Supplementary Figs. S1 and S2. These latter findings suggested that, based on gustatory signals, those high in DIF are less confident in their expectations about their future interoceptive sensations.

### Interoceptive coherence (IC)

A Drink (glucose/sucralose) × DIF (covariate) univariate ANOVA was conducted with interoceptive coherence (IC) as the DV. The DIF × Drink interaction was not significant (*F* (1,58) = 2.644, *p* = 0.109, η^2^ = 0.044). However, there was a significant main effect of DIF (*F* (1,58) = 5.004, *p* < 0.029, η^2^ = 0.079); those with higher DIF had a stronger correlation between blood glucose levels and subjective hunger (*r* = 0.258, p < 0.043; Fig. 4: bottom right). These findings suggested that those with higher DIF are more sensitive to changes in postprandial blood glucose. IC may indicate a higher sensory sensitivity in this domain. See also Table 1 for zero order correlations between DIF and each of the interoceptive indices, which suggested that the main effect of DIF may be driven primarily by the positive association in the glucose condition.

### Rebound hunger: interoceptive prediction error (IPE)

To determine whether those with DIF experienced greater rebound hunger after a sensory incongruent drink a Drink (glucose/sucralose) × Time (0, 15, 30, 60 min) × DIF (covariate) ANCOVA was conducted. The Time × Drink × DIF interaction was significant (*F* (3, 174) = 6.320, *p* = 0.001, η^2^ = 0.098). Follow up tests revealed that after 30 (*r* = 0.531, *p* < 0.002) and 60 (*r* = 0.381, *p* < 0.034) minutes those who consumed sucralose were hungrier. However, this effect was only evident in those high in DIF (Fig. 4: top left). This finding suggested that those high in DIF may be more reactive to the surprising interoceptive state produced by the flavour-nutrient ‘mismatch’ in the sucralose condition. Therefore, rebound hunger may be a useful index of sensory reactivity in the eating domain.

### Glucose tolerance

To determine whether those with DIF have poorer glucose tolerance a Drink (glucose/sucralose) × Time (0, 15, 30, 60 min) × DIF (covariate) ANCOVA was conducted. The Time × Drink × DIF interaction was significant (*F* (3, 174) = 5.921, *p* = 0.001, η^2^ = 0.093). To probe this interaction, a median split was used to dichotomise DIF (High DIF/Low DIF). Follow up t-tests revealed that in the glucose condition, those high in DIF had higher blood glucose after 60 min (*r* = 0.571, *p* < 0.001). All other effects were not significant (all *p* > 0.1). Blood glucose levels did not vary according to DIF in the sucralose condition. These findings suggest that those with DIF have difficulty regulating their blood glucose levels after a glucose load.

### Sweetness and liking

A Drink (glucose/sucralose) × DIF (covariate) ANCOVA determined that participants found both drinks similarly sweet (Glucose 48.6 (5.1), Sucralose 50.0 (5.1): *F* (1, 58) = 0.022, *p* = 0.884, η^2^ = 0.000). In addition, subjective sweetness was not associated with DIF (*F* (1,58) = 0.955, *p* = 0.333, η^2^ = 0.016), and the interaction DIF × Drink was also not significant (*F* (1,58) = 0.046, *p* = 0.830, η^2^ = 0.001). When the analysis was repeated for drink liking, ratings were similar for both drinks (Glucose 61.8 (5.1), Sucralose 56.2 (5.1): *F* (1, 58) = 0.201, *p* = 0.655, η^2^ = 0.003). The interaction DIF × Drink was also not significant (*F* (1,58) = 0.047, *p* = 0.829, η^2^ = 0.001). However, those with DIF reported significantly less liking for the drinks overall (*F* (1,58) = 5.921, *p* < 0.018, η^2^ = 0.093).

### Summary

Overall, the results suggested that those with DIF were characterised by low prior precision and increased sensory reactivity in this domain. The consistent pattern of results demonstrates the efficacy of the present approach for identifying individuals driven by sensations rather than prior beliefs.

## Associations with BMI

DIF is often associated with disordered eating and having a higher BMI ^12^. However, studies assessing the association between interoception and BMI have been inconsistent ^19,59^. The present approach may shed light on this discrepancy. Therefore, it was considered whether the BMI of the present participants was associated with the interoceptive indices. Data are presented in Table 1 and Supplementary Tables S1 and S2. DIF was associated with having a higher BMI in both conditions: Glucose (*r* = 0.360, *p* < 0.047), and Sucralose (*r* = 0.367, *p* < 0.043). Those with a higher BMI were also less confident in their expected satiety in both conditions: Glucose (*r* =  − 0.503, *p* < 0.004), and Sucralose (*r* =  − 0.441, *p* < 0.013). A higher BMI was also associated with a larger satiety divergence but only in the glucose condition (*r* =  − 0.463, *p* < 0.009). Interestingly, in the sucralose condition a higher BMI was also associated with a greater tendency for hunger to be related to changes in blood glucose (*r* = 0.410, *p* < 0.022; Table 1), although this was not significant when the absolute correlation was considered (*r* = 0.054, *p* = 0.774) (Supplementary Table S1). These findings suggested that the interoceptive deficits in those with a higher BMI may be due to a lower prior precision.

To determine whether the aforementioned associations between the interoceptive indices and DIF were explained by differences in BMI, we examined the partial correlations between DIF and the interoceptive indices whilst controlling for BMI. Results are presented in Supplementary Table S2. Generally, the pattern of results remained the same, however the effect size for satiety expectation confidence after tasting was reduced. This suggests that low expected satiety confidence might be a common factor in those with a higher BMI and DIF.

## Discussion

Interoception is recognised to play a role in disordered eating ^16,37^. However, recent advances in our understanding of brain functioning ^3^ demanded the refining of existing paradigms. For the first time the present study took an active inference perspective to understanding the processes underlying satiety. A paradigm was developed that was able to identify individuals who are driven by prior beliefs about future interoceptive states, rather than incoming sensations (Figs. 1, 2, 3, 4). The general pattern of relationships between key variables in this paradigm were consistent with the active inference framework, although some effects sizes were small and effects only reached significant after the sensory congruent drink (Table 1). Importantly, the paradigm was able to discern individual differences in the interoceptive processes underlying satiety in those with DIF, demonstrating the efficacy of the approach (Fig. 4). Specifically, those with DIF had a larger divergence between their expected and actual satiety. In addition, those with DIF lacked confidence in their satiety expectations and instead were driven by changes in physiology (Fig. 4). These findings are important as they show that it is possible to elucidate individual differences in specific processes underlying satiety, thereby resolving key methodological and conceptual challenges in the interoception literature.

An important observation in the present study was that after participants consumed a sensory congruent drink, satiety divergence (SD) and expected satiety confidence (ESC) were inversely associated with the correlation between changes in hunger and changes in blood glucose (interoceptive coherence; IC) (Table 1). This supports the idea that individuals who are driven strongly by expectations are less influenced by changes in physiology and vice versa. Additionally, here we observed that a higher ESC was associated with a smaller divergence between expected and actual satiety (Table 1). Therefore, those who are more confident in their satiety expectations are more likely to assimilate these into their postprandial feeling of satiety. These findings are consistent with the predictive coding notion that the relative influence of ascending sensations, and descending prior beliefs, is governed by their *relative* precision (which may be experienced as subjective confidence) (Fig. 1) ^32^. For the first time it was demonstrated that the approach applies to satiety with important implications for existing paradigms.

For example, over the years a literature has emerged that quantifies interoceptive sensitivity using the satiety quotient (SQ). This had led to the claim that at least 10% of the obese population have a “low satiety phenotype” ^16,36^. However, when considered from the perspective of predictive coding the SQ becomes hard to interpret. From this perspective, subjective postprandial changes (e.g., fullness) might be considered posterior beliefs that result from the assimilation of sensory information and prior beliefs concerning a foods anticipated satiety ^60,61^ (Fig. 1). Therefore, it is unclear exactly what aspect of interoception is deficient in the “low satiety phenotype”. For example, the present data suggested that being overweight might be related to an uncertainty concerning future interoceptive states i.e., low satiety expectation confidence, rather than an inability to detect physiological changes per se (Table 1). This will be an important avenue for future research.

Interestingly, a separate literature has begun to document the expected satiety associated with a range of foods ^62^. This literature has predominantly focused on the food properties influencing expectations, such as the macronutrient content ^63^, energy density ^62^, flavour and viscosity ^64^ and food labelling ^65^. This focus on food characteristics has meant that conclusions were drawn from population averages leaving individual differences relatively neglected. Nonetheless, the present data highlight that aspects of personality, such as alexithymia, and BMI might be important correlates of satiety expectations (Table 1, Fig. 4). However, it should be noted that the present approach differs from that taken previously by those interested in expected satiety. Here we were concerned with the extent to which individuals assimilate prior expectations into their postprandial feeling of satiety, rather than the nature of the expectation per se. Therefore, future research will need to consider individual variation in the nature of satiety expectations, and also their certainty.

Crucially, we were able to demonstrate the present paradigms efficacy by identifying individual differences in the interoceptive processes underlying satiety. Using this method, the pattern of findings is consistent with the view that those with DIF are characterised by low prior precision within the satiety domain. Specifically, those with DIF had a larger discrepancy between their expected and actual satiety (Fig. 4). A consequence is that those with DIF may lack the ability to predict future sensations. Given the important role that satiety expectations play in pre-meal planning and portion size selection ^37^, this finding may help explain the increased prevalence of obesity and disordered eating in those with DIF ^5^. Indeed, we observed that those with a higher BMI were also characterised by a higher satiety divergence in the glucose condition (Table 1). Future research could extend the present paradigm to consider whether this deficit in DIF reflects an inability to construct high-level abstract satiety representations.

A second observation was that those with DIF were less confident in their satiety expectation after tasting the drink (Fig. 4). Importantly, this effect was a consequence of those low in DIF increasing their confidence once they had tasted the drink, while those high in DIF did not. Note that there was no difference in confidence before tasting which suggests that the association with alexithymia after tasting was not driven by overly precise expectations based on visual information. Therefore, although speculative, the finding may reflect a failure to contextualise precision estimation based on new gustatory evidence. From a psychological perspective, precision estimation can be considered a metacognitive process, whereby in an uncertain and changeable world one must continually infer not only the probable change in sensory signals, but also their relative reliability. Indeed, through computational modelling it was recently shown that a reduced recognition of context results in low expected information gain and diminished attentional processing of that sensory channel ^66^. In other words, if those with DIF have an a priori expectation that taste does not convey reliable information concerning satiety, they would be less likely to attend to and rely on this information.

To our knowledge, only one study has assessed gustatory sensitivity in those with DIF. It was reported that those high in alexithymia had poorer salty taste discrimination—an effect that could not be explained by poorer perceptual (colour) discrimination per se ^9^. Together with the present study, these findings suggest that those with DIF may not have updated their confidence after tasting the drink because they may have expected unreliable gustatory information that undermined their subsequent confidence. It will be important for future research to extend the present paradigm to include a psychophysical measure of gustatory sensitivity to elucidate further these processes.

One ramification of weak prior precision is that high-level generalizable representations will be replaced by a reliance on (potentially noisy) bottom-up sensory input (Fig. 1). Indeed, in the present study those with DIF had a stronger correlation between changes in blood glucose and changes in hunger (Fig. 4). These data suggested that those high in DIF give a disproportionate weight to bottom-up internal sensations in driving their postprandial appetitive sensations. Regarding eating behaviour, it has been argued that interoceptive hypersensitivity may lead to positive alliesthesia for food cues, thereby undermining appetite control ^67^. Again, this may help explain the increased levels of disordered eating and obesity in those with alexithymia ^5^.

Importantly, an increased sensitivity to physiological changes may also increase the likelihood of experiencing “rebound hunger”. ‘Rebound hunger’ tends to be observed when a consumed food/drink produces a “mismatch” between orosensory and interoceptive signals. For example, when participants consumed a ‘satiety enhanced’ (thick and creamy) low calorie drink, they felt hungrier compared to when they had consumed the same beverage without these sensory enhancements ^68^. Here we capitalised on this effect to determine whether it could be used to identify individuals who are more likely to respond subjectively to unexpected interoceptive states. Interestingly, we observed that those with DIF were hungrier thirty and sixty minutes after consuming sucralose (a sweet taste that provides no calories) (Fig. 4). The same effect was not observed after participants high in DIF consumed glucose (a sweet taste that provides calories), or in those low in DIF (Fig. 4).

The finding that those high in DIF may be particularly sensitive to ‘rebound hunger’ is important for a number of reasons. Firstly, it demonstrates that phenomenon such as ‘rebound hunger’ may be useful for identifying populations with deficient or exaggerated processing of internal signals. For example, in the future this approach could be used to examine sensory responsivity in those with eating disorders such as anorexia nervosa or bulimia nervosa. Secondly, these findings suggest that when it comes to the development of “sensory enhanced” foods that are designed to “trick” consumers into believing a food will be filling ^55^, individual differences will need to be considered. Specifically, only some individuals—those driven by expectations—are likely to benefit from such interventions.

Finally, when it comes to glucoregulatory sensitivity these data suggest that those with DIF may in fact be more sensitive. This is in contrast to previous research which has associated alexithymia with poor interoceptive sensitivity in other domains e.g., low heartbeat counting accuracy ^15,23^, albeit with inconsistent results ^69^. One explanation for this inconsistency is that, as explained in the introduction, the Heartbeat Counting Task confounds sensory sensitivity and prior beliefs about heartrate ^34,70^. It is therefore possible that previous reports of low heartbeat counting accuracy in alexithymia might reflect differences in expectations about how many beats will be felt, rather than a sensitivity to the heartbeat. Alternatively, those with DIF may be hypersensitive to glucoregulatory signals while lacking sensitivity in other domains. Nonetheless, the overall pattern of results from the present study suggests that self-reported DIF might reflect an inability to predict and contextualise internal states, rather than the tendency to not sense or respond to them at all. Given the novelty of this suggestion more data are required, however, if correct this would have significant implications for the way disorders associated with DIF, such as eating disorders and obesity, are treated.

The limitations of this approach should be considered. Firstly, although the majority of the associations between key variables were consistent with the active inference framework, some were not. In particular, the non-significant relationship between IC and SD after tasting in the sucralose condition is surprising. Although the sample size was similar to, or larger than, those used in similar lines of research ^2,9,39,44,48,49^, this could represent a type two error. Nonetheless, even if this were the case, the size of the coefficients (− 0.164 to − 0.268) would indicate only small to medium sized effects. Satiety is a multifaceted process including numerous neural, metabolic and hormonal signals, of which blood glucose is only one. Therefore, it is possible that other interoceptive signals e.g., ghrelin, CCK, GLP-1, and PYY concentrations might also need to be measured to gain a more accurate understanding of active inference in the context of satiety. From a predictive coding perspective, when afferent sensations are noisy or volatile their precision is reduced, thereby reducing their influence on perception ^71^. Therefore, it is possible that after consuming artificial sweetener participants may switch to a reliance on other interoceptive signals. This also has potential ramifications for the habitual consumption of artificial sweetener, which may render internal signals chronically unpredictable leading to high expected uncertainty, particularly when consumed in combination with caloric sweeteners ^72^. This should be determined in future research.

A second limitation is the use of VAS to measure expected satiety and confidence. VAS are sensitive to between food differences, easily compared with postprandial satiety, and ecologically valid (i.e., people rated and then consumed real food items) ^46^. However, they are potentially subject to bias e.g., trait over/under confidence and/or interpretational bias. An alternative approach might be paradigms based on the method of constant stimuli (MCS) ^62^. However, the MCS has traditionally been used to compare group differences in satiety expectations between foods ^62^, rather than to examine individual differences. Nonetheless, the calculation of an individual difference measure (Weber’s fraction) could facilitate the identification of differences in the ability to discriminate the satiating properties of different foods. However, such tasks would need to be further developed to determine whether individuals who can more precisely discriminate the satiating properties of foods are more likely to be driven by this information when making inferences about their interoceptive state.

Concerning our interoceptive coherence measure, it was interesting to note that in some participants the correlation coefficient between changes in blood glucose and changes in hunger was positive (i.e. higher levels of hunger despite raising blood glucose levels). Conceptually, a strong positive correlation between hunger and blood glucose may be different from a strong negative correlation, and more research is needed to understand the interoceptive processes leading to such disparate subjective experiences. Finally, we chose to focus on the DIF scale of alexithymia as this component is most often associated with eating behaviour ^5^. However, it is possible that results may differ if other components of alexithymia e.g., Difficulty Describing Feelings or Externally Oriented Thinking, were considered. Future research should consider this possibility.

In conclusion, a novel interoception paradigm is reported that was able to elucidate individual differences in the propensity to be driven by satiety expectations versus internal sensations. Previous work has highlighted the individual importance of satiety expectations ^37^, postprandial hunger and satiety ^16^, and humoral signals ^73^. However, studying these processes individually has limited our understanding of the possible inter-relationships between key concepts. Taking an active inference approach, we found that individuals vary in the extent to which they rely on prior expectations and afferent signals when determining their postprandial satiety. Specifically, those who reported difficulties identifying feelings (a component of alexithymia: DIF) lacked confidence in their expected satiety and had a larger divergence between their expected and postprandial satiety. Conversely, those with DIF had a greater correspondence between changes in hunger and changes in blood glucose, and experienced greater rebound hunger after an artificially sweetened drink, indicating a reactive rather than prospective regulatory style. These findings, along with recent advances in our understanding of brain functioning ^3^, indicate the need to better appreciate individual variation in the way key processes underling interoception interact. Further research building on the present paradigm has the potential to pave the way towards more successful personally tailored psychological and nutritional interventions.

## Supplementary Information

Supplementary Information.
