# Publisher Correction: Individual differences in sensory and expectation driven interoceptive processes: a novel paradigm with implications for alexithymia, disordered eating and obesity

**DOI:** 10.1038/s41598-021-96996-z

**Published:** 2021-09-08

**Authors:** Hayley A. Young, Chantelle M. Gaylor, Danielle de‑Kerckhove, David Benton

**Affiliations:** Department of Psychology, Swansea University, Wales, SA2 8PP UK

Correction to: *Scientific Reports* 10.1038/s41598-021-89417-8, published online 12 May 2021

The original version of this Article contained errors.

In the PDF version, Table 1 was incorrectly sized resulting in the loss of the final two columns of data: “DIF” and “BMI”. The HTML version was unaffected.

In addition, there was a repeated error in the *F* statistics, where *F* (1, 58) and *F* (3, 174) were incorrectly given as *F* = (1, 58) and *F* = (3, 174) respectively.

The original Article has been corrected.

The conclusions of the Article remain unchanged.

