# Supplementary material for: Individual differences in sensory and expectation driven interoceptive processes: a novel paradigm with implications for alexithymia, disordered eating and obesity
Source: Sci Rep. 2021 May 12;11:10065. doi: 10.1038/s41598-021-89417-8 (PMC8115295; doi:10.1038/s41598-021-89417-8)
Supplement: Supplementary file 1 — Supplementary Information. [file 41598_2021_89417_MOESM1_ESM.docx]

**Individual Differences in Sensory and Expectation Driven Interoceptive**

**Processes: A Novel Paradigm with implications for Alexithymia,**

**Disordered Eating and Obesity**

Hayley A Young*, Chantelle M Gaylor, Danielle de- Kerckhove and David Benton

**Manipulation check**

After consuming the entire beverage participants evaluated how sweet they found the drink, how much they liked the drink, its visual appeal, its mouth-feel, its odour, initial taste, and after taste using 100mm visual analogue scales (VAS). There were no significant differences between the drinks on any of the dimensions: Sweetness (*F* = (1, 60) = 1.093, *p* =0.300; Glucose 57.6(29.3), Sucralose 50.1(27.2)), Liking (*F* = (1, 60) = 0.184, *p* =0.669; Glucose 62.1(29.2), Sucralose 58.9(19.2)), Mouth-feel (*F* = (1, 60) = 0.237, *p* =0.629; Glucose 60.9(24.4), Sucralose 56.7(36.0), Visual appeal (*F* = (1, 60) = 0.351, p =0.556; Glucose 60.7(30.3), Sucralose 55.5(31.6)), Odour (*F* = (1, 60) = 0.036, *p* =0.850; Glucose 65.3(23.3), Sucralose 64.0(26.5)), Initial taste (*F* = (1, 60) = 1.406, *p* =0.241; Glucose 60.7(30.3), Sucralose 55.5(31.6))), and After taste (*F* = (1, 60) = 1.180, *p* =0.283; Glucose 58.6(23.4), Sucralose 49.7(34.4)).

**Supplementary Figure 1**

*Change in Expected Satiety Confidence in those with High or Low Difficulty Describing Feelings.*


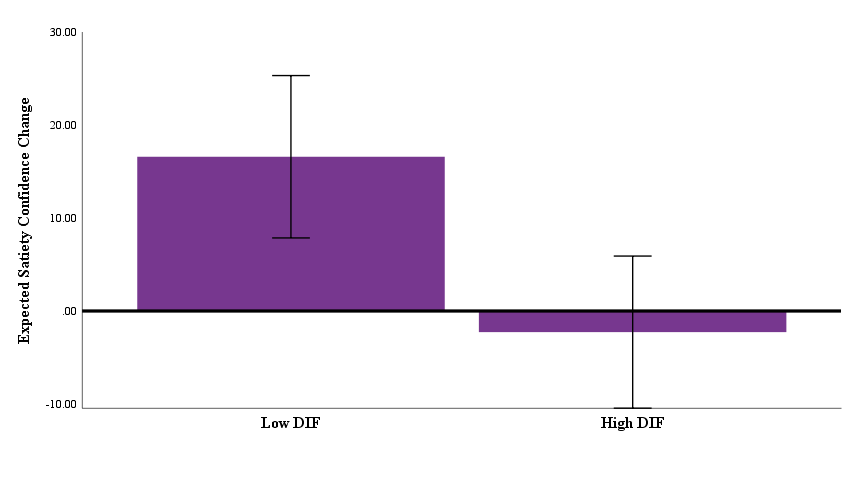


*Note.* Error bars are 95% confidence intervals. A median split was used to dichotomise Difficulty Identifying Feelings (DIF). Change in Expected Satiety Confidence (ESC) is ESC after tasting the drink minus ESC before tasting the drink. Whereas those with low DIF increased their ESC after taking the drink, those with high DIF did not (*F* = (1, 60) = 9.945, *p* <0.003, η2 =0.142).

**Supplementary Figure 2**

*Change in Expected Satiety Confidence Before and After Tasting in those with High or Low Difficulty Describing Feelings.*


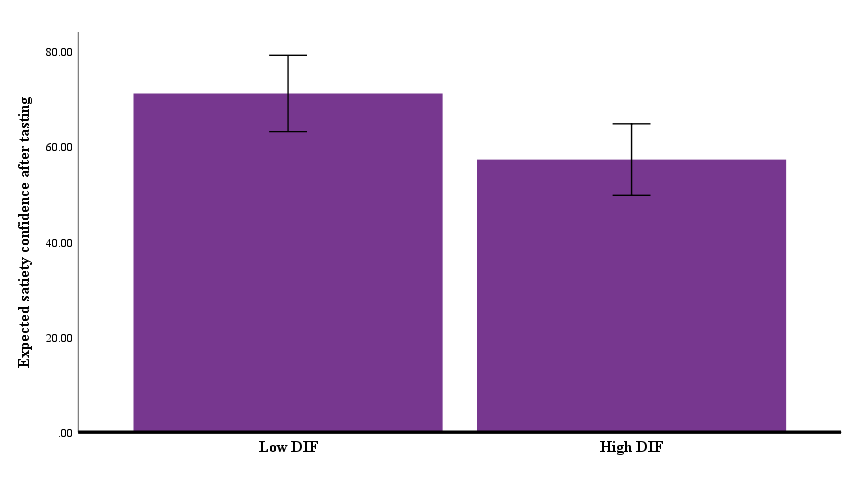

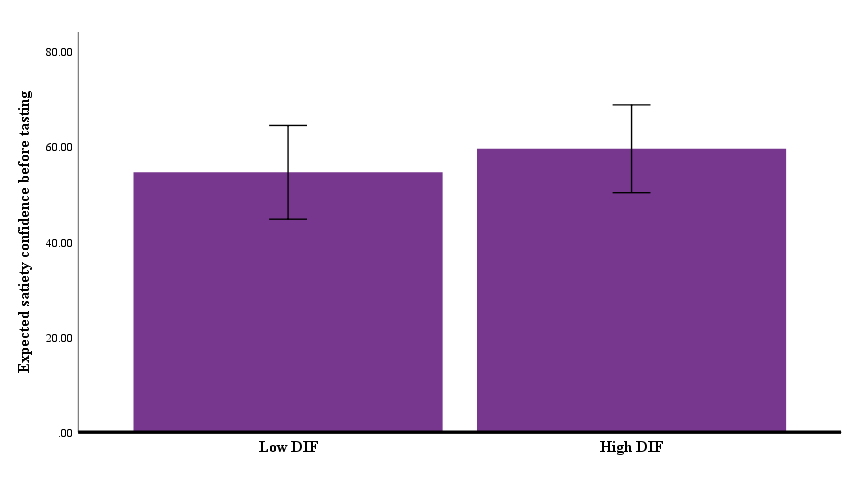


*Note. .* Error bars are 95% confidence intervals. A median split was used to dichotomise Difficulty Identifying Feelings (DIF). Expected Satiety Confidence did not differ between those high or low in DIF before tasting the drink (*F* = (1, 60) = 0.536, *p* =0.467, η2 =0.009). However, after tasting the drink those low in DIF were more confident in predicting their future satiety (*F* = (1, 60) = 6.402, *p* <0.014, η2 =0.016).

**Supplementary Table 1.**

*Zero Order Correlations between the Interoceptive Indices, Alexithymia, and BMI in the Glucose and Sucralose Conditions.*

| GLUCOSE | | | | | | | SUCRALOSE | | | | | | |
| --- | --- | --- | --- | --- | --- | --- | --- | --- | --- | --- | --- | --- | --- |
|  | **ESC-AT** | **SD-BT** | **SD-AT** | **IC (ABS)** | **DIF** | **BMI** |  | **ESC-AT** | **SD-BT** | **SD-AT** | **IC (ABS)** | **DIF** | **BMI** |
| ESC-BT | .503* | .407* | -.027 | -.399* | -.123 | -.108 | **ESC-BT** | .428* | .267 | -.096 | -.506* | .276 | .053 |
| ESC-AT |  | .482** | .550** | -.501** | -.370* | -.503** | **ESC-AT** |  | .114 | .280 | -.261 | -.325* | -.441* |
| SD-BT |  |  | .604** | -.331* | -.671** | -.328 | **SD-BT** |  |  | .386* | -.437* | .249 | -.094 |
| SD-AT |  |  |  | -.232 | -.713** | -.463** | **SD-AT** |  |  |  | -.067 | -.094 | -.229 |
| IC (ABS) |  |  |  |  | .435* | .174 | **IC (ABS)** |  |  |  |  | -.182 | .054 |
| DIF |  |  |  |  |  | .360* | **DIF** |  |  |  |  |  | .367* |

*Note.* N = 62. Red – significant positive correlation, Purple – significant negative correlation. * p<0.05, **p<0.001. IC – Interoceptive Coherence, SD – Satiety Divergence, ESC – Expected Satiety Confidence, BT – Before Tasting, AT – After Tasting, DIF – Difficulty Identifying Feelings, BMI – Body Mass Index. IC-ABS - Interoceptive Coherence Absolute (in this analysis interoceptive coherence was taken as the absolute Pearson’s r correlation between hunger and blood glucose levels - rather than the signed Pearson’s r correlation that is reported in the main analysis).

**Supplementary Table 2.**

*Partial Correlations between the Interoceptive Indices and Alexithymia controlling for BMI in the Glucose and Sucralose Conditions.*

| GLUCOSE | | | | | | | SUCRALOSE | | | | | | |
| --- | --- | --- | --- | --- | --- | --- | --- | --- | --- | --- | --- | --- | --- |
|  | **ESC-AT** | **SD-BT** | **SD-AT** | **IC**  **(ABS)** | **IC** | **DIF** |  | **ESC-AT** | **SD-BT** | **SD-AT** | **IC**  **(ABS)** | **IC** | **DIF** |
| ESC-BT | .522** | .396* | -.026 | -.389* | -.260 | -.090 | **ESC-BT** | .504** | .273 | -.086 | -.510** | -.309 | .276 |
| ESC-AT |  | .389* | .414* | -.486** | -.535** | -.234 | **ESC-AT** |  | .081 | .204 | -.265 | -.107 | -.195 |
| SD-BT |  |  | .540** | -.295 | -.317 | -.628** | **SD-BT** |  |  | .376* | -.434* | -.227 | .306 |
| SD-AT |  |  |  | -.174 | -.339* | -.661** | **SD-AT** |  |  |  | -.056 | -.079 | -.011 |
| IC (ABS) |  |  |  |  | .711** | .406* | **IC (ABS)** |  |  |  |  | .403* | -.217 |
| IC |  |  |  |  |  | .519** | **IC** |  |  |  |  |  | -.091 |

*Note.* N = 62. Red – significant positive correlation, Purple – significant negative correlation. * p<0.05, **p<0.001. IC – Interoceptive Coherence, IC (ABS) – Interoceptive Coherence Absolute, SD – Satiety Divergence, ESC – Expected Satiety Confidence, BT – Before Tasting, AT – After Tasting, DIF – Difficulty Identifying Feelings, BMI – Body Mass Index.
